# Supplementary material for: Generation of raptor diversity in Europe: linking speciation with climate changes and the ability to migrate
Source: PeerJ. 2022 Dec 8;10:e14505. doi: 10.7717/peerj.14505 (PMC9745957; doi:10.7717/peerj.14505)
Supplement: Supplemental Information 1 [file peerj-10-14505-s001.pdf]

Supplementary Material

Generation of raptor diversity in Europe: linking speciation with climate changes and the ability to migrate

Juan J. Negro[1], Eduardo Rodríguez-Rodríguez[2], Airam Rodríguez[3,4,5], Keith Bildstein[6]

[1]Department of Evolutionary Ecology, Estación Biológica de Doñana-CSIC, Sevilla, Spain.

[2]Department of Integrated Sciences, Faculty of Experimental Sciences, University of Huelva, Huelva, Spain.

[3]Grupo de Ornitología e Historia Natural de las islas Canarias (GOHNIC), Buenavista del Norte, Canary Islands, Spain.

[4]Terrestrial Ecology Group (TEG-UAM), Department of Ecology, Universidad Autónoma de Madrid, Madrid, Spain.

[5]Centro de Investigación en Biodiversidad y Cambio Global (CIBC-UAM), Universidad Autónoma de Madrid, Madrid, Spain.

[6]116 Village Drive, Blandon, PA 19510, USA.

Content

Table S1. Factor loadings and importance of the components of the principal components analysis (PCA) on fourteen morphometric variables.

Table S2. Trophic niche variables used in the analysis of trophic niche.

Code. *R* scripts for the cluster dendrogram and K-means analysis.

Figure S1. Groups resulting from the K-means cluster analysis with 16 centers. The groups are composed by the same species as in Fig. 1 (main text). Cluster stability (measured as average Jaccard distances) is also included after species names.

Figure S2. Pairwise correlations of the first principal component (body size index) and seven morphometric variables (i.e. those representing the lowest values of the ranges of each morphometric). Black numbers indicate Pearson correlation coefficients and red stars indicate if the correlation is highly significant (*** < 0.001).

Figure S3. European raptors grouped according to both trophic niche and phylogenetic relationships to show morphological and plumage similarities.

Table S1

	PC1	PC2	PC3	PC4	PC5
Factor loadings					
Size_HBW1[a]	-0.276	0.136	-0.029	0.311	-0.084
Size_HBW2[a]	-0.275	0.088	-0.096	0.190	0.499
Wingspan_HBW1[a]	-0.273	0.103	0.425	-0.061	-0.156
Wingspan_HBW2[a]	-0.261	0.032	0.721	-0.413	0.089
Length1[b]	-0.278	0.050	-0.024	0.241	-0.181
Length2[b]	-0.277	0.087	-0.117	0.084	0.240
Wingspan1[b]	-0.276	0.067	0.119	0.397	-0.196
Wingspan2[b]	-0.276	0.076	0.090	0.341	0.216
Tail1[b]	-0.253	0.429	-0.249	-0.215	-0.465
Tail2[b]	-0.249	0.437	-0.345	-0.477	0.202
Weight_M1[b]	-0.263	-0.374	-0.107	-0.080	-0.158
Weight_M2[b]	-0.260	-0.381	-0.155	-0.096	-0.419
Weight_F1[b]	-0.263	-0.349	-0.146	-0.233	0.239
Weight_F2[b]	-0.260	-0.401	-0.123	-0.103	0.147
Importance of components					
Standard deviation	3.563	0.891	0.472	0.314	0.252
Proportion of variance	0.907	0.057	0.016	0.007	0.005
Cumulative proportion	0.907	0.964	0.979	0.986	0.991

Table S1. Factor loadings and importance of the components of the principal components analysis (PCA) on 14 morphometric variables. [a] and [b] taken from Del Hoyo et al. (1994) and Ferguson-Lees & Christie (2001), respectively.

Table S2

Species	Predator	Scavenger	fish	small birds	large birds	small mammals	Large mammals	insects	herps	Scavenging type	Foraging method
Pandion haliaetus	yes	no	yes	no	no	no	no	no	no	no	foraging on water
Haliaeetus albicilla	yes	no	yes	no	yes	no	yes	no	no	no	foraging on water
Elanus caeruleus	yes	no	no	yes	no	yes	no	yes	yes	no	open foraging
Circaetus gallicus	yes	no	no	no	no	no	no	no	yes	no	perch hunting
Pernis apivorus	yes	no	no	no	no	no	no	yes	no	no	ground hunting
Circus aeruginosus	yes	yes	yes	yes	yes	yes	no	no	no	small pieces	foraging over wetlands
Circus pygargus	yes	no	no	yes	no	yes	no	yes	yes	no	open foraging close to ground
Circus cyaneus	yes	no	no	yes	no	yes	no	yes	yes	no	open foraging close to ground
Circus macrourus	yes	no	no	yes	no	yes	no	yes	yes	no	open foraging close to ground
Accipiter gentilis	yes	no	no	yes	yes	yes	no	no	no	no	forest ambush
Accipiter brevipes	yes	no	no	yes	yes	yes	no	no	no	no	forest ambush
Accipiter nisus	yes	no	no	yes	yes	yes	no	no	no	no	forest ambush
Milvus migrans	yes	yes	no	yes	no	yes	no	yes	yes	small pieces	open foraging
Milvus milvus	yes	yes	no	yes	no	yes	no	yes	yes	small pieces	open foraging
Buteo buteo	yes	yes	no	yes	yes	yes	no	no	yes	small pieces	perch hunting
Buteo lagopus	yes	yes	no	yes	yes	yes	no	no	yes	small pieces	perch hunting
Buteo rufinus	yes	yes	no	yes	yes	yes	no	no	yes	small pieces	perch hunting
Hieraaetus pennatus	yes	no	no	no	yes	no	yes	no	yes	no	open foraging
Aquila fasciata	yes	no	no	no	yes	no	yes	no	yes	no	open foraging
Aquila chrysaetos	yes	yes	no	no	yes	no	yes	no	yes	Large pieces	open foraging
Aquila adalberti	yes	yes	no	no	yes	no	yes	no	yes	Large pieces	open foraging
Aquila heliaca	yes	yes	no	no	yes	no	yes	no	yes	Large pieces	open foraging
Clanga clanga	yes	yes	no	no	yes	yes	yes	no	yes	Large pieces	open foraging
Clanga pomarina	yes	yes	no	no	yes	yes	yes	no	yes	Large pieces	open foraging
Aegypius monachus	no	yes	no	no	no	no	no	no	no	Large pieces	open foraging
Gyps fulvus	no	yes	no	no	no	no	no	no	no	Large pieces	open foraging
Gypaetus barbatus	no	yes	no	no	no	no	no	no	no	small pieces	open foraging
Neophron percnopterus	no	yes	no	no	no	no	no	no	no	small pieces	open foraging
Falco naumanni	yes	no	no	yes	no	yes	no	yes	yes	no	open foraging
Falco vespertinus	yes	no	no	yes	no	yes	no	yes	yes	no	open foraging
Falco tinnunculus	yes	no	no	yes	no	yes	no	yes	yes	no	open foraging
Falco eleonorae	yes	no	no	yes	yes	yes	no	no	yes	no	perch and aerial hunting
Falco subbuteo	yes	no	no	yes	yes	yes	no	no	yes	no	perch and aerial hunting
Falco peregrinus	yes	no	no	yes	yes	yes	yes	no	yes	no	perch and aerial hunting
Falco columbarius	yes	no	no	yes	yes	yes	yes	no	yes	no	perch and aerial hunting
Falco biarmicus	yes	no	no	yes	yes	yes	yes	no	yes	no	perch and aerial hunting
Falco rusticolus	yes	no	no	yes	yes	yes	yes	no	yes	no	perch and aerial hunting
Falco cherrug	yes	no	no	yes	yes	yes	yes	no	yes	no	perch and aerial hunting

Table S2. Behavioural and dietary data used to generate trophic niche groups (from Del Hoyo et al. 1994 and Fergusson-Lees and Christie 2001).

Code

***R* scripts**

```
## Upload data
library(readxl)
x <- read_excel("TableS2.xlsx")
x <- as.data.frame(x)

## Data preparation
x[x == "yes"] <- "1"
x[x == "no"] <- "0"

x$ScavengingSmallPieces <- x$Scavengingtype
x$ScavengingSmallPieces[x$ScavengingSmallPieces == "small pieces"] <- "1"
x$ScavengingSmallPieces[x$ScavengingSmallPieces == "Large pieces"] <- "0"

x$ScavengingLargePieces <- x$Scavengingtype
x$ScavengingLargePieces[x$ScavengingLargePieces == "small pieces"] <- "0"
x$ScavengingLargePieces[x$ScavengingLargePieces == "Large pieces"] <- "1"

x$fonwater <- x$Foragingmethod
x$fonwater[x$fonwater != "foraging on water"] <- "0"
x$fonwater[x$fonwater == "foraging on water"] <- "1"

x$openf <- x$Foragingmethod
x$openf[x$openf != "open foraging"] <- "0"
x$openf[x$openf == "open foraging"] <- "1"

x$ph <- x$Foragingmethod
x$ph [x$ph != "perch hunting"] <- "0"
x$ph [x$ph == "perch hunting"] <- "1"

x$gh <- x$Foragingmethod
x$gh [x$gh != "ground hunting"] <- "0"
x$gh [x$gh == "ground hunting"] <- "1"

x$fowet <- x$Foragingmethod
x$fowet [x$fowet != "foraging over wetlands"] <- "0"
x$fowet [x$fowet == "foraging over wetlands"] <- "1"

x$ofcg <- x$Foragingmethod
x$ofcg [x$ofcg != "open foraging close to ground"] <- "0"
x$ofcg [x$ofcg == "open foraging close to ground"] <- "1"

x$fa <- x$Foragingmethod
x$fa [x$fa != "forest ambush"] <- "0"
x$fa [x$fa == "forest ambush"] <- "1"
```

```
x$ah <- x$Foragingmethod
x$ah [x$ah != "perch and aerial hunting"] <- "0"
x$ah [x$ah == "perch and aerial hunting"] <- "1"

x1 <- x[,c(2:10, 13:22)]
x1[ , 1:19] <- as.data.frame(apply(x1[ , 1:19], 2, as.numeric))
```

## cluster dendrogram

```
## First we obtain the binary distance Matrix
mat_distb <- dist(x = x1, method = "binary")

## Now, we generate the cluster ##
labs = x$Species
hc_binary_av <- hclust(mat_distb, method = "complete")

## And Finally we plot it ##

plot(hc_binary_av, labels= labs, font=3, main="")
```

## K-means cluster analysis

```
library(factoextra)
k16 <- kmeans (x1, centers = 16, nstart = 25)
str (k16)
k16

x1[ , 1:19] <- as.data.frame(apply(x1[ , 1:19], 2, as.numeric))
fviz_cluster(k16, data = x1, repel = T, ggtheme = theme_bw() +
               theme(panel.grid.major = element_blank(),
               panel.grid.minor = element_blank(),
               axis.line = element_line(colour = "black")))
```

## Bootstrap the results to check for cluster adequacy

```
library(fpc)
clusters <- 16
groups <- cutree(hc_binary_av, k=clusters)
clus.boot <- clusterboot(x1, B = 1000, clustermethod = hclustCBI,
      method = "ward.D", k = clusters, count = FALSE)
clus.boot$bootmean
x1$hierarchical_clusters <- as.factor(groups)
set.seed(8675309)
AvgJaccard <- clus.boot$bootmean
Instability <- clus.boot$bootbrd/1000
Clusters <- c(1:clusters)
Eval16 <- as.data.frame(cbind(Clusters, AvgJaccard, Instability))
Eval16
```

Figure S1

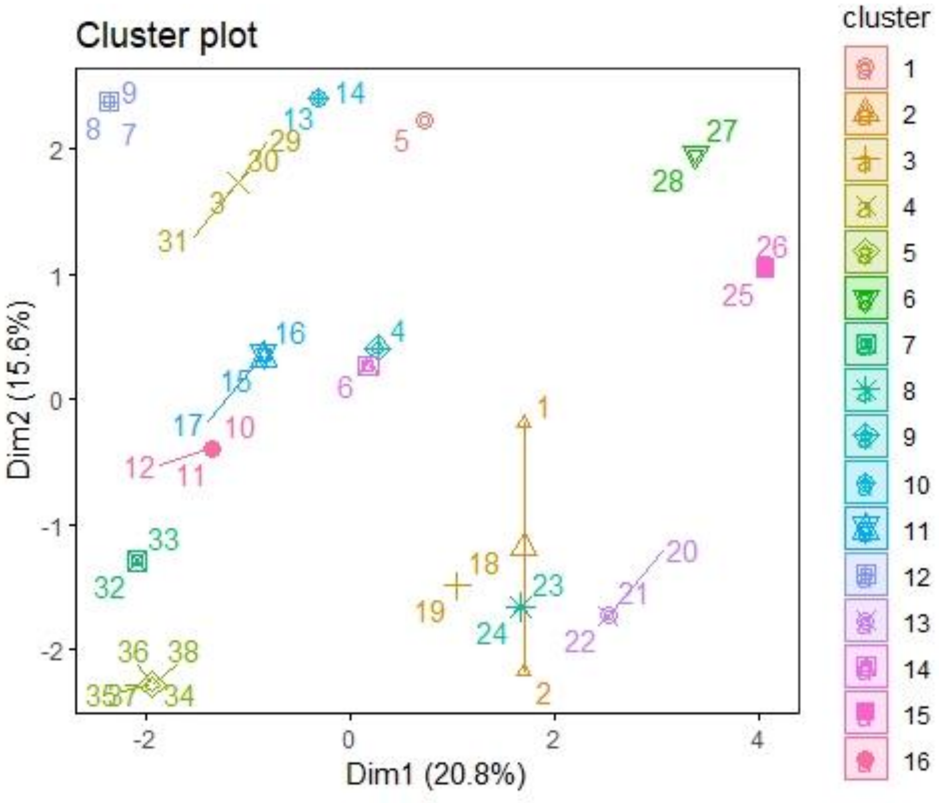

Figure S1. Groups resulting from the K-means cluster analysis with 16 centers. Cluster stability (measured as average Jaccard distances) is also included after species names.

Numbers, species and clusters

Number	Species	Cluster
1	*Pandion haliaetus*	1
2	*Haliaeetus albicilla*	2
3	*Elanus caeruleus*	3
4	*Circaetus gallicus*	4
5	*Pernis apivorus*	5
6	*Circus aeruginosus*	6
7	*Circus pygargus*	7
8	*Circus cyaneus*	7
9	*Circus macrourus*	7
10	*Accipiter gentilis*	8
11	*Accipiter brevipes*	8
12	*Accipiter nisus*	8
13	*Milvus migrans*	9
14	*Milvus milvus*	9
15	*Buteo buteo*	10
16	*Buteo lagopus*	10
17	*Buteo rufinus*	10
18	*Hieraaetus pennatus*	11
19	*Aquila fasciata*	11
20	*Aquila chrysaetos*	12

21	*Aquila adalberti*	12
22	*Aquila heliaca*	12
23	*Clanga clanga*	12
24	*Clanga pomarina*	12
25	*Aegypius monachus*	13
26	*Gyps fulvus*	13
27	*Gypaetus barbatus*	14
28	*Neophron percnopterus*	14
29	*Falco naumanni*	3
30	*Falco vespertinus*	3
31	*Falco tinnunculus*	3
32	*Falco eleonorae*	15
33	*Falco subbuteo*	15
34	*Falco peregrinus*	16
35	*Falco columbarius*	16
36	*Falco biarmicus*	16
37	*Falco rusticolus*	16
38	*Falco cherrug*	16

Cluster Stability

As a rule of thumb, average Jaccard distance < 0.6 indicates cluster is unstable. Average Jaccard distance > 0.85 indicates cluster is highly stable.

Clusters	Average Jaccard	Instability
1	0.689	0.489
2	0.893	0.155
3	0.644	0.356
4	0.652	0.348
5	0.624	0.376
6	0.883	0.171
7	0.883	0.178
8	0.818	0.232
9	0.875	0.180
10	0.817	0.240
11	0.867	0.194
12	0.800	0.257
13	0.833	0.215
14	0.830	0.214
15	0.820	0.235
16	0.919	0.093

Figure S2

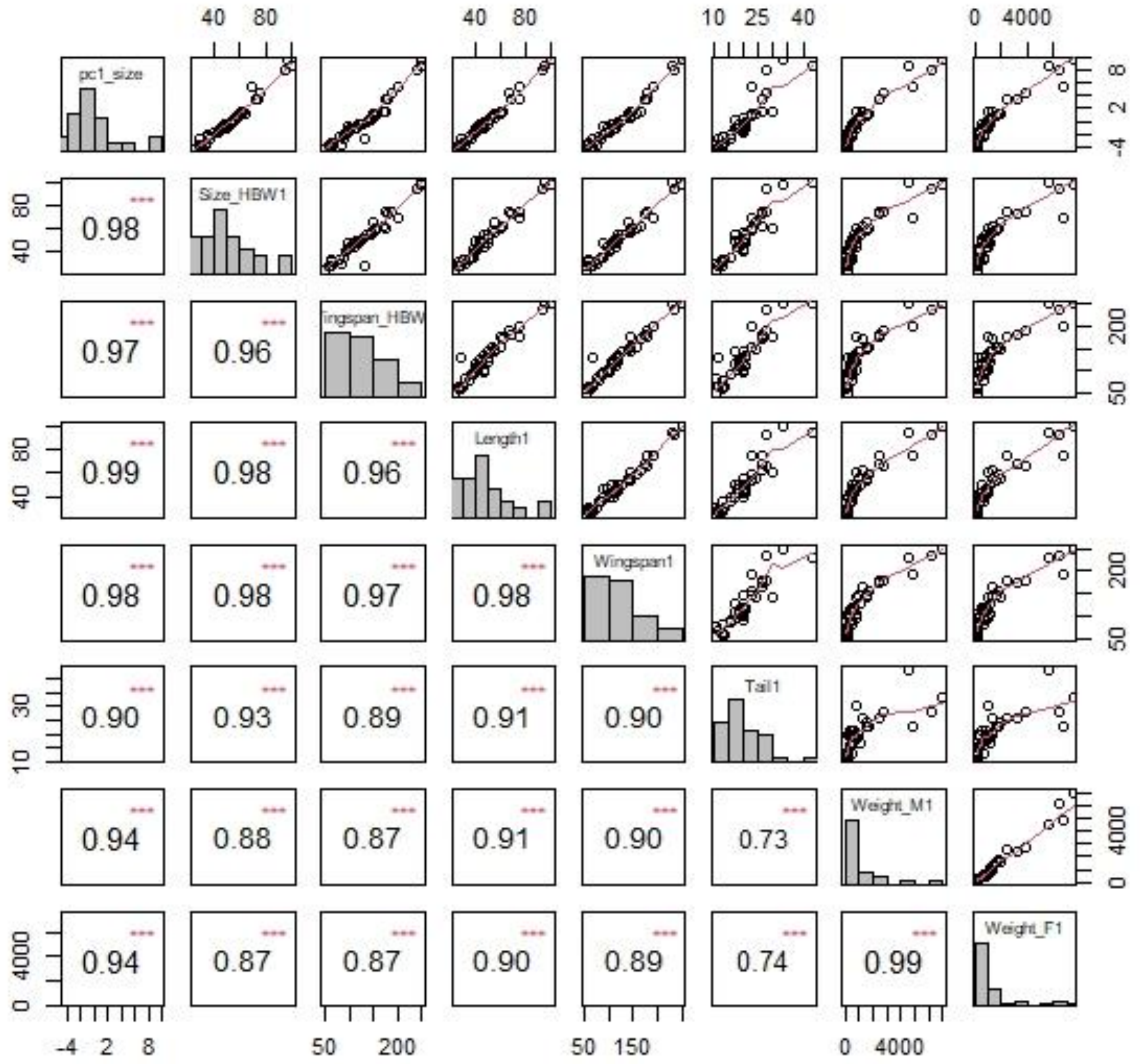

Figure S2. Pairwise correlations of the first principal component (body size index) and seven morphometric variables (i.e. those representing the lower ranges of each morphometric). Black numbers indicate Pearson correlation coefficients and red stars indicate if the correlation is highly significant (*** < 0.001).

Figure S3

Group 1: *Haliaeetus albicilla* (left) by J.J. Negro. *Pandion haliaetus* (right) by J.A. Irastorza.

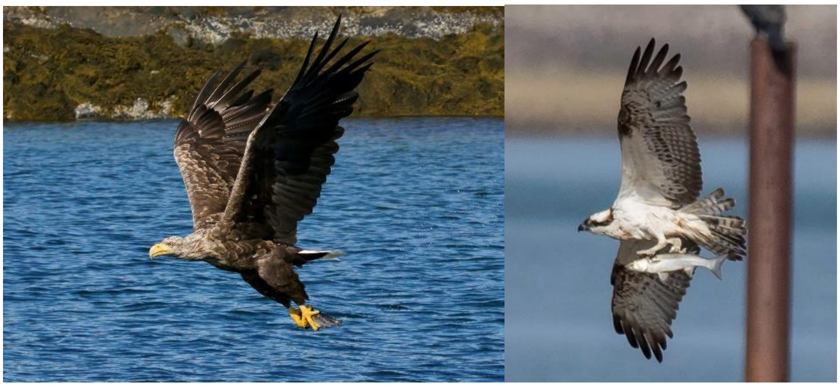

Group 2: *Circus macrourus* (left) by J.J. Negro. *C. pygargus* (center) by J.J. Negro. *C. cyaneus* (right) by B. Rodríguez.

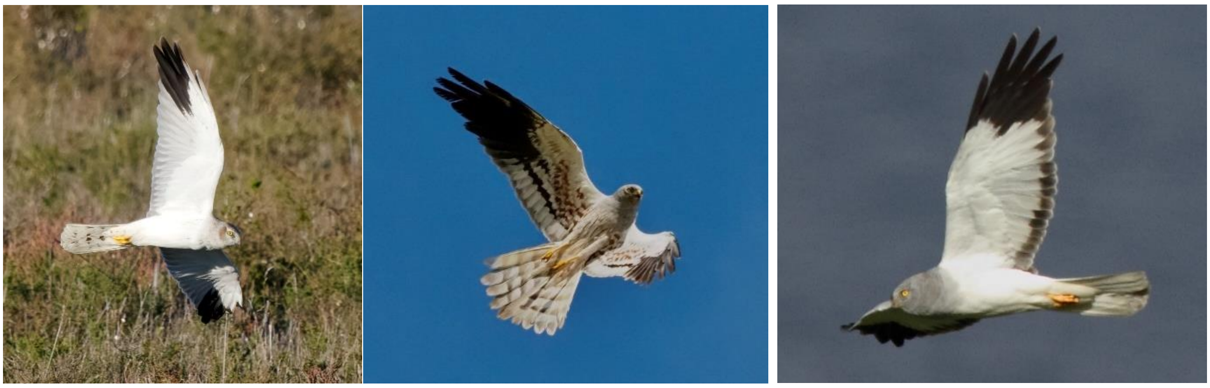

Group 3: *Accipiter gentilis* (left) and *A. nisus* (center) by M. Cayuela. *A. brevipes* (right) by V. Y. Arkhipov (Creative Commons).

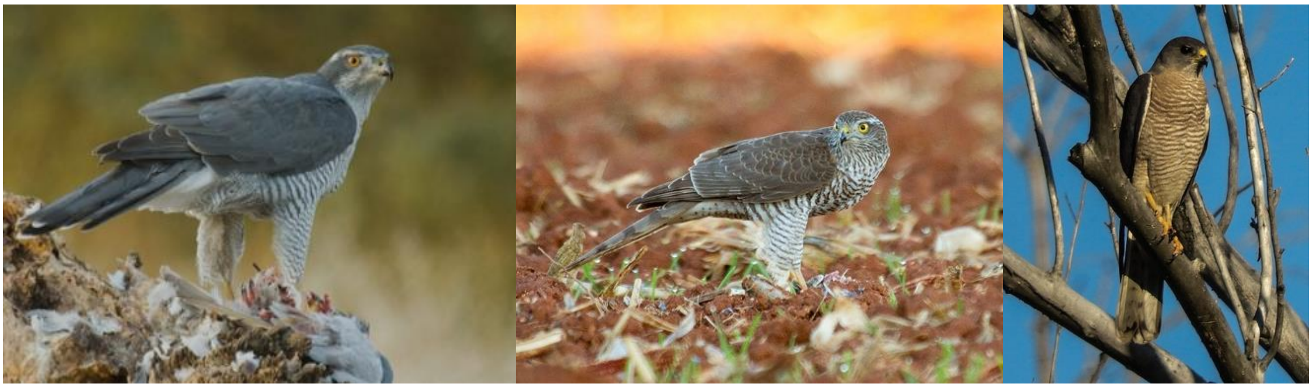

Group 4: *Milvus milvus* (left) and *M. migrans* by J.J. Negro.

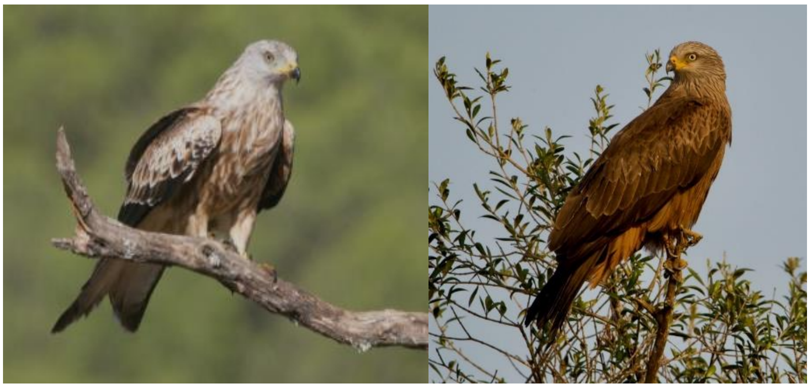

Group 5: *Buteo lagopus* (left) by M. Szczepanek (Creative Commons). *B. buteo* (center) by E.J. Rodríguez-Rodríguez. B. *rufinus* (right) by K. Koshy (Creative Commons).

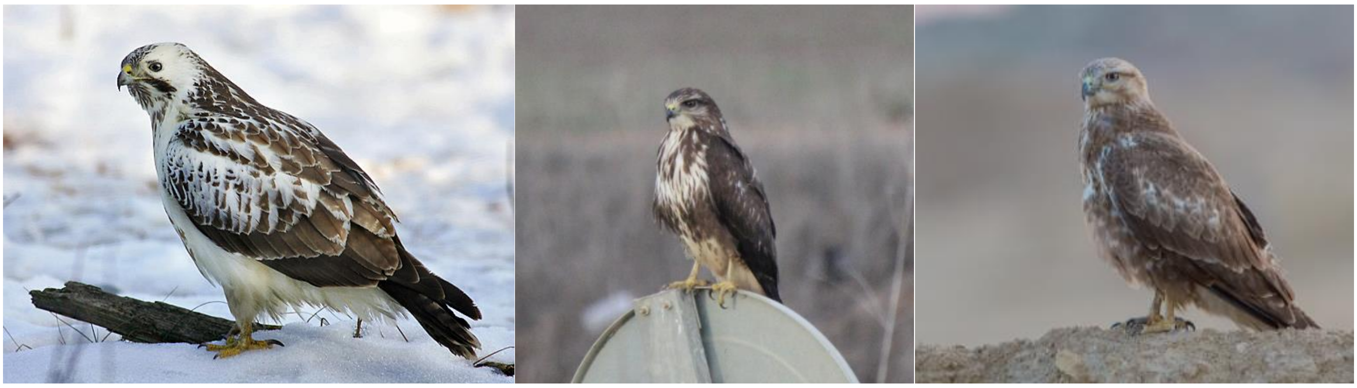

Group 6: *Aquila fasciata* (left) by J.A. Irastorza. *Hieraaetus pennatus* (right) by J.J. Negro.

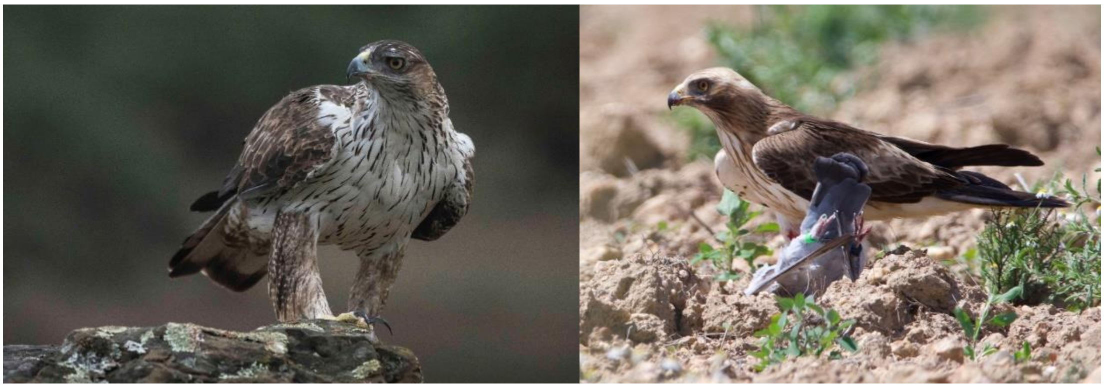

Group 7: *Aquila chrysaetos* (left) and *A. adalberti* (center) by J.J.Negro. *A. heliaca* (right) by A. Kovacs.

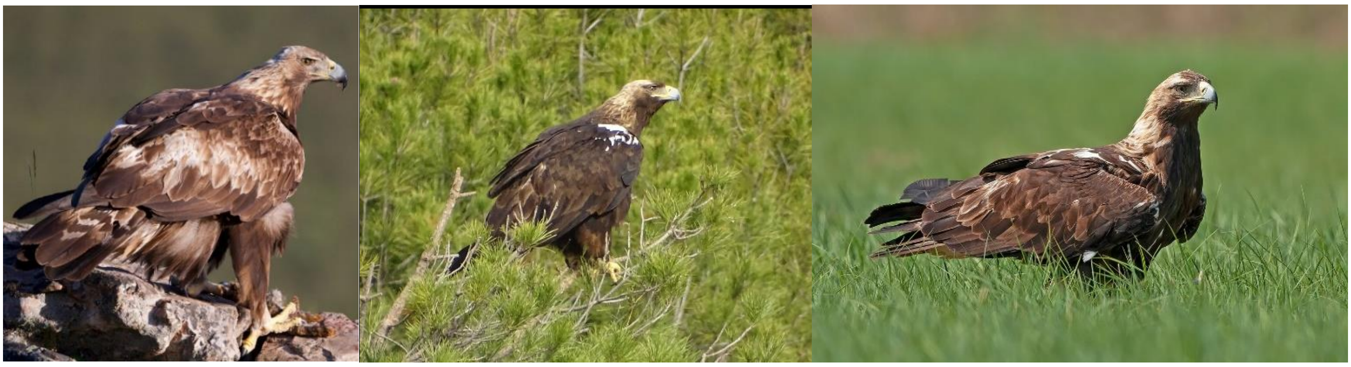

Group 8: *Clanga clanga* (left) by A. Kovacs. *C. pomarina* (right) by W. Avnerunder (Creative Commons). Juvenile plumage (both).

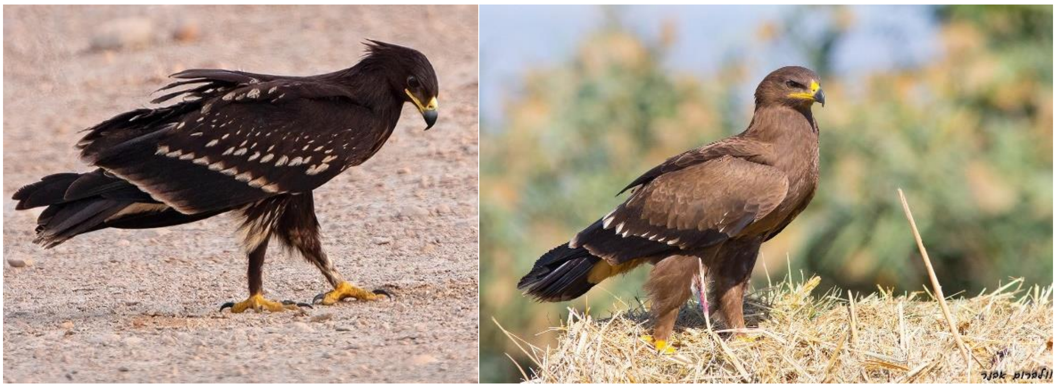

Group 9: *Gyps fulvus* (left and center) *and Aegypius monachus* (right) by J.J. Negro.

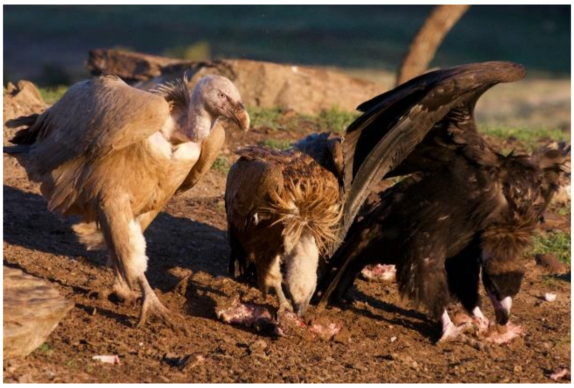

Group 10: *Gypaetus barbatus* (left) and *Neophron percnopterus* (right) by J.J. Negro.

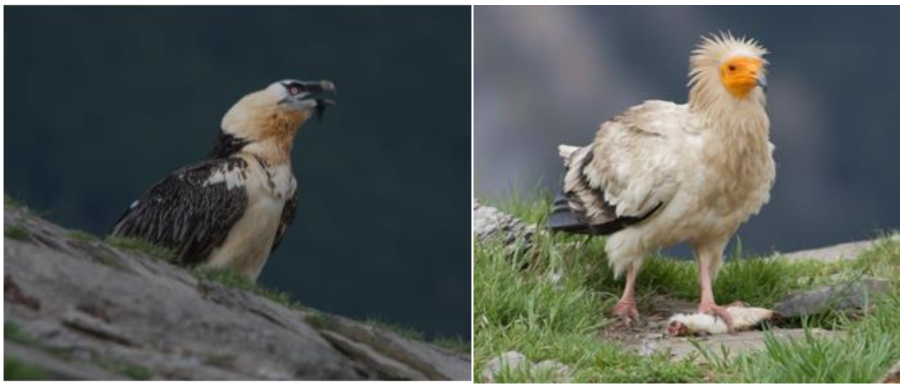

Group 11: *Falco eleonorae* (left) by B. Rodríguez. *F. subbuteo* (right) by J.J. Negro.

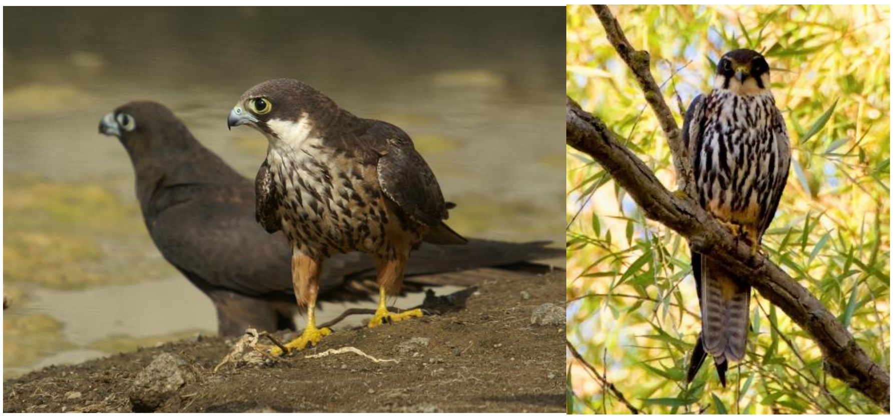

Group 12: *Falco rusticolus* (left) by NorthernLight (Creative Commons). *F. cherrug* (center) by B. Rodríguez. *F. peregrinus* (right) by J.M. Sayago. *F. biarmicus* (right second row) by D. Keats (Creative Commons). *F. columbarius* by J.J. Negro.

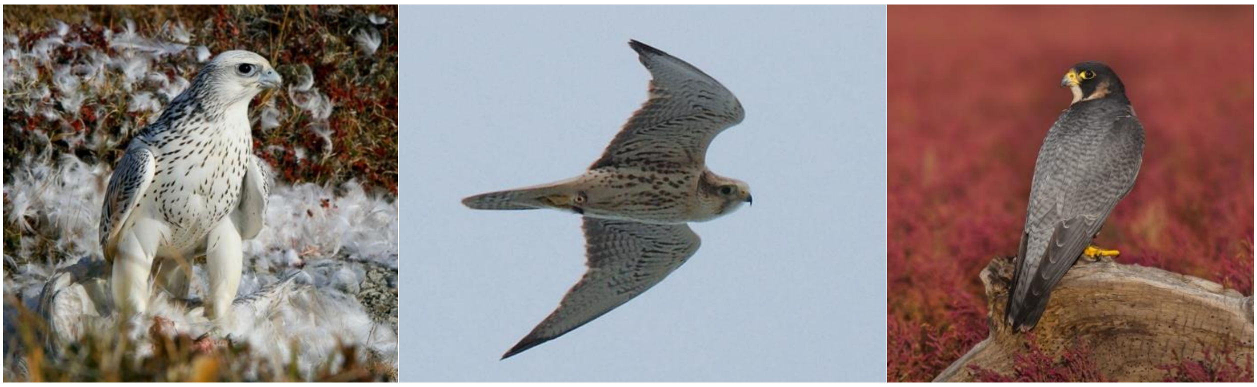

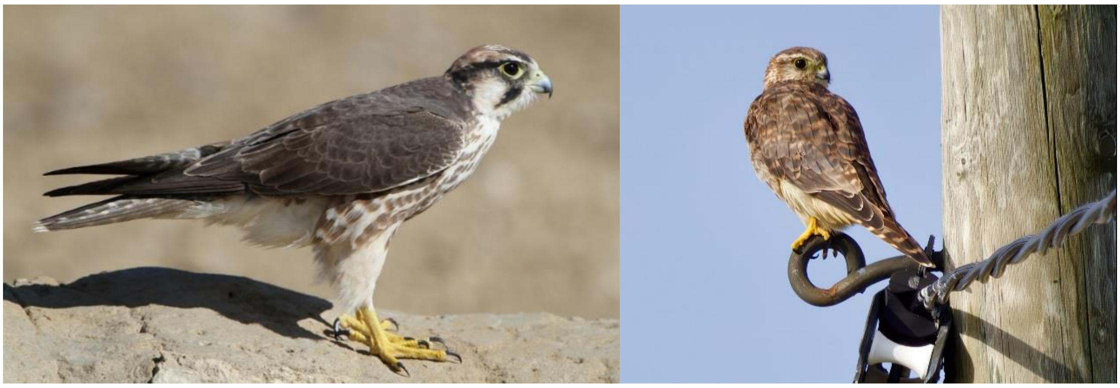

Group 13: *Falco tinnunculus* (left) and *F. naumanni* (center) by J.J. Negro. *F. vespertinus* (right) by B. Rodríguez.

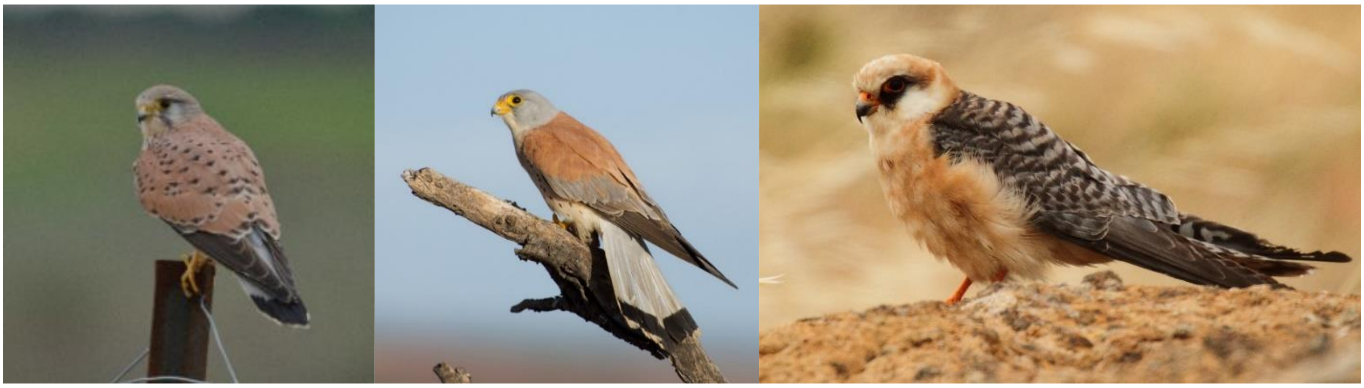

Figure S3. European raptors grouped according to trophic niche and phylogenetic relationships to show morphological and plumage similarities.

Creative Commons attributions

-*Accipiter brevipes* by Vladimir Yu. Arkhipov (https://commons.wikimedia.org/wiki/Special:Contributions/Arkhivov) under CC BY_SA 4.0 (https://creativecommons.org/licenses/by-sa/4.0/deed.en)

-*Buteo lagopus* by Marek Szczepanek (https://commons.wikimedia.org/wiki/User:Pkuczynski/Marek_Szczepanek) under CC BY_SA 3.0 (https://creativecommons.org/licenses/by-sa/3.0/legalcode)

-*Buteo rufinus* by Koshy Koshy (https://www.flickr.com/people/97235261@N00) under CC BY 2.0 (https://creativecommons.org/licenses/by/2.0/legalcode)

-*Clanga pomarina* by Wolbrun Avner (Creative Commons Attribution-ShareAlike 4.0 International)

-*Falco rusticolus* by NorthernLight (https://de.wikipedia.org/wiki/Benutzer:NorthernLight) under CC BY_SA 3.0 (https://creativecommons.org/licenses/by-sa/3.0/legalcode)

-*Falco biarmicus* by Derek Keats (https://www.flickr.com/people/93242958@N00) (https://es.wikipedia.org/wiki/Falco_biarmicus#/media/Archivo:Lanner_falcon,_Falco_biarmicus,_at_Kgalagadi_Transfrontier_Park,_Northern_Cape,_South_Africa_(34447024731).jpg) under CC BY 2.0 (https://creativecommons.org/licenses/by/2.0/legalcode)
